# Crave-Out: A Distraction/Motivation Mobile Game to Assist in Smoking Cessation

**DOI:** 10.2196/games.4566

**Published:** 2016-05-26

**Authors:** Kathryn L DeLaughter, Rajani S Sadasivam, Ariana Kamberi, Thomas M English, Greg L Seward, S Wayne Chan, Julie E Volkman, Daniel J Amante, Thomas K Houston

**Affiliations:** ^1^ University of Massachusetts Medical School Worcester, MA United States; ^2^ CHOIR ENRM VAMC Bedford, MA United States; ^3^ University of Massachusetts Medical School Quantitative Health Sciences Worcester, MA United States; ^4^ University of Massachusetts Medical School Psychiatry Worcester, MA United States; ^5^ Bryant University Department of Health Communication Smithfield, RI United States

**Keywords:** smoking cessation, Internet, secondary prevention, health behavior

## Abstract

**Background:**

Smoking is still the number one preventable cause of death. Cravings—an intense desire or longing for a cigarette—are a major contributor to quit attempt failure. New tools to help smokers’ manage their cravings are needed.

**Objective:**

To present a case study of the development process and testing of a distraction/motivation game (Crave-Out) to help manage cravings.

**Methods:**

We used a phased approach: in Phase 1 (alpha testing), we tested and refined the game concept, using a Web-based prototype. In Phase 2 (beta testing), we evaluated the distraction/motivation potential of the mobile game prototype, using a prepost design. After varying duration of abstinence, smokers completed the Questionnaire of Smoking Urge-Brief (QSU-Brief) measurement before and after playing Crave-Out. Paired *t* tests were used to compare pregame and postgame QSU-Brief levels. To test dissemination potential, we released the game on the Apple iTunes App Store and tracked downloads between December 22, 2011, and May 5, 2014.

**Results:**

Our concept refinement resulted in a multilevel, pattern memory challenge game, with each level increasing in difficulty. Smokers could play the game as long as they wanted. At the end of each level, smokers were provided clear goals for the next level and rewards (positive reinforcement using motivational tokens that represented a benefit of quitting smoking). Negative reinforcement was removed in alpha testing as smokers felt it reminded them of smoking. Measurement of QSU-Brief (N=30) resulted in a pregame mean of 3.24 (SD 1.65) and postgame mean of 2.99 (SD 1.40) with an overall decrease of 0.25 in cravings (not statistically significant). In a subset analysis, the QSU-Brief decrease was significant for smokers abstinent for more than 48 hours (N=5) with a pregame mean of 2.84 (SD 1.16) and a postgame mean of 2.0 (SD 0.94; change=0.84; *P* =.03). Between December 22, 2011, and May 29, 2014, the game was downloaded 3372 times from the App-Store, with 1526 smokers visiting the online resource www.decide2quit.org linked to the game.

**Conclusions:**

Overall, playing the game resulted in small, but nonsignificant decreases in cravings, with changes greater for those had already quit for more than 48 hours. Lessons learned can inform further development. Future research could incorporate mHealth games in multicomponent cessation interventions.

**Trial Registration:**

Clinicaltrials.gov NCT00797628; https://clinicaltrials.gov/ct2/show/NCT00797628 (Archived by WebCite at http://www.webcitation.org/6hbJr6LWG)

## Introduction

Smoking is the number one preventable cause of death [1]. In the United States alone, each year over 480,000 deaths are due to smoking, including from second hand smoke [2]. While the majority of smokers want and have tried to quit, quitting for good is challenging. After quit attempts, relapse rates are as high as 90% [3,4]. While combination FDA-approved pharmacotherapy and behavioral interventions can assist during quit attempts, cravings, an intense desire or longing for a cigarette [5], are a major contributor to quit attempt failure [6]. Most smokers report experiencing cravings or urges to smoke while quitting [7]. Because cravings play a major role in quit attempt failure [8], additional tools to help smokers manage cravings are needed.

Behavioral techniques such as substitution, relaxation, and distraction have been used to help smokers manage cravings [9]. Cravings can occur at any time and are often triggered by a cue, which could be anything from persons, places, things, or mood states. Thus, craving management tools should be readily available at the point of need [10]. The proliferation of mobile phones, and the increasing number of users who play games on their mobile phones presents a new opportunity to design point-of-need relapse prevention tools [11]. Games on a mobile phone can be engaging and distracting, and smokers could play them until their cravings subside. While any mobile game may help distract smokers, a game specifically designed for cravings could also remind smokers of reasons for quitting and for remaining quit. Developing games for health is challenging as they should be educational, incorporate evidenced-based behavioral strategies, and be engaging and fun [12]. Can a fun, challenging distraction/motivation game be developed to help smokers manage cravings?

**Figure 1:**
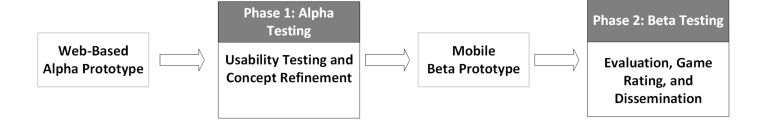
Game development phases.

We developed and pilot tested a mobile phone game—Crave-Out—for distracting smokers during a craving and motivating them by reinforcing the benefits of quitting. Consistent with the goals of a pilot study [13-17], this small-scale study was designed to assess the feasibility of the game development process, evaluate users’ experience in game play, and measure changes in urges to smoke, an important targeted behavioral process. As recommended by game developers [18-21], we developed Crave-Out by using a mixed-methods approach. This study collected qualitative and quantitative data and included two phases (alpha and beta testing; Figure 1):

- Phase 1: Alpha testing (usability testing and concept refinement) encompassed the predevelopment phase in which the game concept was refined using a prototype. Game concepts are crucial to the success of the game; they determines what type of a game it is, how much fun it is, and how much it supports the goals of the game [22]. Prototypes are a recommended approach for concept testing because they collect the thoughts of the users by providing a window into how they interact with the game [22].
- Phase 2: During beta testing (evaluation and dissemination), we developed a mobile version of the game based on the refined concept and evaluated it using a prepost change in cravings assessment. We also tested whether smokers were satisfied with the game and thought it was fun. We also tested the potential of disseminating the game on the Apple iTunes App Store [23], Apple’s official repository for downloading apps for the iPhone and iPad.

Since the methods of Phase 2 depended on the results of Phase 1, to improve readability, we first describe the methods and results of Phase 1, followed by the methods and results of Phase 2. Finally, we summarized the main findings of these two studies in the discussion. Our paper demonstrates how an iterative, user-driven pilot testing approach can be used to develop a game. Our protocol was approved by the University of Massachusetts Medical School Institutional Review Board.

## Phase 1: Alpha Testing (Usability Testing and Concept Refinement)

To refine the distraction/motivation concept, we tested the alpha version of our game. The results of concept refinement informed the development of our beta version. We describe the methods and results of our alpha testing below.

### Phase 1: Methods

To develop our prototype for concept refinement, we adapted an existing Web-based game that required clicking on pictures to win points. Our intent was to create a distraction tool. On the basis of classical conditioning, including the counterpurpose and relapse prevention model, we redesigned the existing Web-based game to reinforce the benefits of quitting by having smokers click on good things associated with quitting or negative things associated with smoking. We enlisted the aid of students to create artwork, and over about 4 1-hour meetings created images to use for the game. We then enlisted an additional high school programmer to develop the game on the Web-based platform. The development of the game took approximately 30 hours to complete. During game play, participants started the game by selecting whether during play they wanted to click on “bad” things associated with smoking (eg, cigarettes and yellow teeth), or “good” things associated with quitting (eg, money, health, and time with family; see Multimedia Appendix A). In each version, there was reinforcement for clicking: If a good thing was clicked, the smoker received positive feedback (a green tick mark and a pleasant sound). Similarly, if a bad thing was clicked, the smoker received negative feedback (display of a red cross mark and a harsh sound).

We tested our concept with current or prior smokers aged 19 years or older recruited using flyers, from the University of Massachusetts Medical School campus and UMass Medical Memorial Center. Flyers were distributed across the campus and a coordinated effort with Department of Preventive Medicine was made to have the flyers available in waiting areas and specifically on posted boards at a connected treatment center where counseling for tobacco and other drugs of use was held. In addition, word of mouth was utilized by contact with tobacco treatment specialists on the staff at the University of Massachusetts Medical School. Participants were asked to report to the usability laboratory to complete participation. We used a think aloud protocol [24-26]: while the participants were playing the game, we asked them to vocalize their thoughts, feelings, and opinions about the game. The think aloud protocol provides information about how a user approaches the interface and what they have in their minds when utilizing the interface. We conducted usability testing with 5 users to test our game per existing recommendations regarding think aloud protocol testing [27], which indicate that the majority of issues can be identified with 4±1 users, each with progressively diminishing returns. After playing the game, participants answered a survey and provided open-ended comments. There are several approaches for evaluating qualitative data, including thematic analysis, narrative summary, and grounded theory [28]. Because one of primary goals is to describe the processes of playing the game, we used a combination of thematic summary and narrative summary. This involved an open coding process (without a predefined set of codes) to develop themes.

### Phase 1: Results

#### Prototype Testing

Out of the 5 Phase 1 participants, 3 were between the ages of 19 and 24, with a median number of cigarettes per day of 5. All 5 participants had tried to quit and relapsed. Smokers made comments on the following themes:

- Engagement: Participants indicated that the game was fun and distracting. One participant commented that he “got into” the game, while another participant commented that he was “curious” about the game. Participants also felt that the game should be expanded with additional scenarios to make it more interesting.
- Positive reinforcements: All the participants indicated that the positive reinforcement was beneficial. Example comments included “the game gave reasons but did not necessarily push me” and the game “gave me motivation to quit.” A third participant said that he was considering quitting after playing the game.
- Negative reinforcement: Participant disliked the images of cigarettes and other negative stimuli with the accompanying warning sounds. The participants observed that looking at the bad things associated with smoking, while informational, made them think about smoking.”

In the follow-up survey, all the participants strongly agreed or agreed that the game would help them think about reasons to quit smoking, 4 out of 5 participants agreed that they would recommend the game, and 3 out of 5 strongly agreed or agreed that they would play the game again. Participants disagreed on whether the game would distract them from cravings, but all 5 agreed that it would help them think about reasons to quit smoking.

#### Concept Refinement

We first removed negative reinforcements and designed the game to present smokers a motivational token highlighting a benefit of staying abstinent after each level. We then conceptualized the following functions:

1. Multilevel game with a Crave-Out function: The game was designed with multiple levels of game play. The initial levels were simpler, followed by levels of increasing difficulty. Instead of the traditional end game function, we designed the “Crave-Out” function that allows smokers to choose to end the game after their craving has reduced. The game will not complete until the Crave-Out button is pressed.
2. Pattern memory challenge game: We redesigned the game as a pattern memory challenge game fashioned like the old “Simon” game, in which players must rely on memory of the order of objects, which in this instance was specific fruit to choose or “catch” with a virtual bucket at the bottom of the screen. We chose fruit and the virtual bucket for their simplicity and because they were not related in any way to common triggers to smoking. We chose an unrelated concept for our primary game construct, based on findings from Phase 1. To prevent triggering thoughts of smoking, the fruits-in-the-bucket was chosen as a neutral construct for the pattern recognition game.
3. Only positive motivational reinforcements: In the new version, the participant during game play uses a virtual bucket to move back and forth and catch the “good” objects (fruit) as they fell downward from the top of the screen. (See Multimedia Appendix B) The game started simple and then progressively became more difficult. In the first level, the requirement was to catch one “good” object (one of the five fruits) that is named before the level starts. If the participant avoided the other falling fruit and caught the correct fruit, the level was completed. As the level increased, the number of good objects to catch in sequence also increased, and the speed at which the good object fell also increased. After each level, a motivational token that represents a benefit of quitting was awarded to the participant and was continuously displayed in the form of a colorful sticker or stamp on the upper section of the screen until the participant clicked on the link to go to the next level. Each sticker or stamp included a visual representation of the benefits of quitting and reinforced the many reasons to not smoke. This token appeared in the top center of the screen (eg, a colorful $ sign representing the money saved by not smoking) once the fruit for each level was “caught.” Additional tokens included more time with family and friends, whiter teeth, better health, more leisure time, and a cleaner home (Appendix A). Following each completed level, a feedback screen was displayed to the smokers. This included a summary of the level completed and their total score, as well as all the motivational tokens that they received during game play. On each feedback screen, there were two buttons at the bottom: “Crave-Out” (used to indicate that one has finished playing) and “Decide2quit” (links to an evidence-based Web-assisted tobacco intervention: www.Decide2quit.org). The Crave-Out button took the player to a summary screen that contained total time played and both current and running total, which enabled them to see the “reduced” amount of time for which they had been distracted from cigarettes (Appendix B).
4. Referral to other cessation tools: We also provided a link to the Decide2Quit.org Web-assisted tobacco intervention from the summary page [29,30]. The Decide2Quit link allowed them to register with the Decide2Quit.org Web site. Decide2Quit.org is an evidence-based Web-assisted tobacco intervention that includes information about quitting smoking; secure asynchronous messaging with a certified tobacco treatment specialist; an online support group; and a motivational, pushed-email, tailored messaging system. Decide2Quit.org is designed for both current and former smokers.

## Phase 2: Beta Testing Pil ot Study (Evaluation of Cravings Reduction and Dissemination)

As noted, on the basis of the results of Phase 1, we developed a mobile version of the game by using the Unity3D game development framework [18]. To develop the game, we first developed the 3D artifacts of the various objects (ie, fruits, bucket, and background scenery items). Once these were developed, we had to program the artifacts to respond to various events or interactions of the game player (eg, the randomness of the different fruits, their speeds and frequencies of recurrences and their tumbling effects, the movement of the bucket from side to side and catching of the fruits in response to the game players’ actions, and the appearance of the reward stickers). Another programming action was to end the level if all the fruits were caught in the right sequence. The developed game was then exported to multiple platforms, including the iPhone and iPad mobile platform, for evaluation using a prepost design. We describe the methods and results of our beta testing below.

### Phase 2: Methods

#### Study Design

Our Phase 2 pilot study employed a mixed-methods (qualitative and quantitative) convergent prepost design including measurement of cravings before and after playing the game [31]. Consistent with the goals of a pilot study, our study was designed to provide (1) data on intervention development feasibility and (2) estimates of effect sizes for key measures (cravings) related to smoking cessation. Importantly, our study was designed to provide estimates of effect on cravings to guide future studies, and was not designed to have definitive results on this measure [13-17]. Although we do conduct statistical tests, our results are designed to inform more definitive research and should be carefully considered within the context of a pilot.

After providing consent, participants completed a pregame questionnaire to assess their cravings and then were provided the game to play. As in Phase 1, we again used think aloud protocol testing, and recorded each session of game play. Once they finished playing the game for 10 minutes, participants then completed the same questionnaire to assess their cravings. Participants also completed a brief survey about the game after they played it. This survey consisted of questions related to how they liked the game and how challenging they felt it was; it included several open-ended questions. Again, as in Phase 1, we compiled the message content and created themes on the basis of the narrative analysis technique.

Further, to test dissemination, once the Crave-Out version was ready for Phase 3 pilot testing, we released the game on the Apple iTunes App Store [21] and tracked downloads. We also tracked visits and registrations to the Decide2Quit.org Web site from the game.

#### Setting and Sample

Current and former smokers from the University of Massachusetts Medical School campus and UMass Medical Memorial Center were recruited using flyers. Smokers admitted to the hospital were referred by a tobacco treatment specialist through the University of Massachusetts Medical School’s psychiatry department.

As with Phase 1, flyers were distributed across the campus and a coordinated effort with Department of Preventive Medicine was made to have the flyers available in waiting areas and specifically on posted boards at a connected treatment center where counseling for tobacco and use of other drugs was held. In addition, word of mouth was utilized by contact with tobacco treatment specialists on the staff at the University of Massachusetts Medical School. Participants were asked to report to the usability laboratory to complete participation. Some participants were recruited while they were inpatients; for those recruited inpatients, the tobacco treatment specialist who was assigned to meet with the patient would ask if he or she was interested in participating. The tobacco treatment specialist would then contact the project coordinators with information regarding patients interested and provided room number and appropriate time for visiting the patient. The project coordinator would take a hard copy of the survey along with a laptop to play the game to the patient’s room and obtain his or her consent before participation.

We chose to recruit a sample of 30 individuals to test the feasibility of our methods, test game usability, and to generate effects on the outcome of cravings.

#### Measures

We used the Questionnaire of Smoking Urge-Brief (QSU-Brief), a 10-item questionnaire on smoking urges utilizing a 7-point Likert scale for responses to assess cravings [32]. The QSU-Brief is a valid and reliable assessment that has become widely used in the measurement of cravings [32-35]. Factor analyses conducted by Cox et al indicated that this brief assessment showed very good reliability as per Cronbach alpha [37}. After providing consent, participants completed the pregame QSU-Brief questionnaire and then were given instructions and access to play the game. In addition to the QSU-Brief, we used a brief demographic questionnaire, including questions on smoking status, and a brief survey about game playing experience for data collection. Smoking status was assessed before game play. Recommendations from the Society for Research on Nicotine and Tobacco on the need for biochemical verification state that the degree of misclassification is moderated by characteristics of the smoking cessation intervention. The more intensive the intervention is, the higher the potential for misclassification due to social desirability. In line with these recommendations, as our intervention (a cravings game) was of low intensity and also because the goal of the intervention was not to induce cessation but to pilot test, we did not use biochemical verification of smoking status [36,37].

Twenty-four qualitative questions on experience playing the game were developed by our team; 6 of these were open-ended questions (see Appendix C). In addition, we collected verbal feedback provided while participants played the game. Using the Apple iTunes App Store dashboard, we recorded download data for each week of the study.

#### Data Analysis

Following a descriptive analysis, we used paired *t* tests to determine differences between pre- and postgame craving levels. We calculated the change in the craving level by subtracting the pregame craving score of each individual from their postgame craving score, to calculate a mean prepost difference. We also conducted stratified analysis by demographic characteristics, smoking status, and game experiences. Note that for our primary analysis and sensitivity analyses, we have presented *P* values “as-is,” recognizing that the stratified sensitivity analyses represent an instance of multiple comparison testing. The statistical literature review of methods for accounting for multiple comparisons has noted that adjustment is controversial and may be over-conservative in some instances [38-40]. Adjusting for multiple comparisons is highly appropriate in exploratory analyses that are not following a specific research question. For studies where the measures follow a specified research goal, recommendations vary. Although we believe that presenting the *P* values "as-is" provides the reader useful information, we recognize the challenge of interpretation in the setting of multiple comparisons and encourage readers to consider these results in the context of this pilot experiment, not as definitive results. Data analysis was performed using STATA 12, Copyright 1996–2016 StataCorp LP.

### Phase 2: Results

#### Demographics

Participants were 30 smokers. The majority were male (20/30, 67%), between ages 25 and 44 (20/30, 67%), college-educated (21/30, 70%), and abstinent for more than 48 hours (5/30 17%). The mean number of cigarettes smoked per day was 13.8 (SD 10.0, range 0-40). Most had attempted to stop smoking at least once in the past 12 months (17/30, 59%) and were willing to stop smoking (20/30, 67%). See Table 1 below regarding these demographics.

**Table:** 

Variable		N=30	%
**Age**			
	19-24	2	7%
	25-34	8	27%
	35-44	10	33%
	45-54	6	20%
	55-64	2	7%
	65+	2	7%
**Gender**			
	Male	20	67%
	Female	10	33%
**Race and ethnicity**			
	White/Non-Hispanic or Latino	30	100%
**Education**			
	Grades 9-11 (some high school)	1	3%
	Grade 12 or GED^a^	8	27%
	College 1-3 years	12	40%
	College 4 years or more	9	30%
**Recruitment source**			
	Outpatient	22	73%
	Inpatient	8	27%
**Ever visited a smoking cessation Web site**			
	Yes	7	23%
	No	23	77%
**Successful quitter (smokers who had 0 cigarettes per day & had stopped smoking in the past 12 months)**			
	Yes	4	23.5%
	No	13	76.5%
**Number of cigarettes smoked per day**			
	None	5	17%
	1-6	4	13%
	10-18	9	30%
	20-25	10	33%
	30-40	2	7%
	Mean number of cigarettes per day	13.8	
	StdSD	10.0	
**Stopped smoking in the past 12 months**			
	Yes	17	59%
	No	13	41%
**Desire to stop smoking**			
	^b^I do not smoke now	5	17%
	Yes	20	67%

^a^GED: General Educational Development

^b^Participants qualified as “do not smoke now” if they had been abstinent for 48 hours or more at the time of the visit.

#### Game Rating

**Figure 2:**
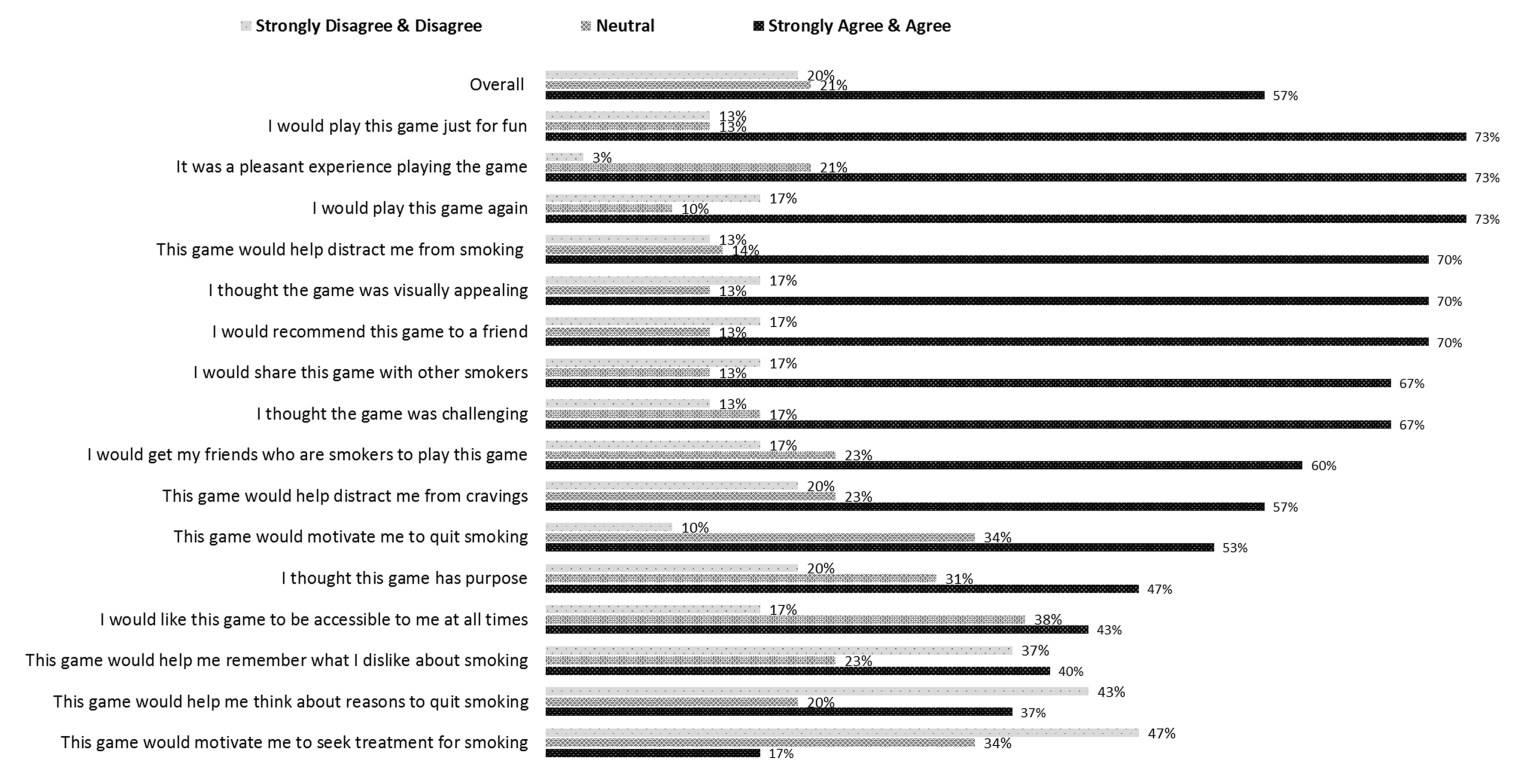
Game experience of Phase 2 smokers.

Smokers reported that Crave-Out was fun (22/30, 73%), challenging (20/30, 67%), and would help distract them from cravings (17/30, 57%; Figure 2). Most also said that they would play the game outside of the study environment (22/30, 73%). The majority of smokers also responded that they found the game would motivate them to quit smoking (16/30, 53%). While playing the game, smokers commented that the game was fun and distracting. Some commented on how they enjoyed playing, for example, “This is amazing,” “I'm just having fun,” and “It takes your mind off smoking.” Most of the smokers played longer than 10 minutes. The following quote indicated one smoker’s desire to continue playing the game, “No, don’t want to quit the game, I just won.” Smokers also noted that the game was challenging. Example comments included “It’s complicated,” “I have to pay attention,” “It’s fast,” “It’s hard,” and “It’s difficult actually.” In addition, one user-interface issue with the beta version was discovered; the 3D effect made it difficult for participants to move the cursor left-right completely accurately in catching the fruit. This was commented on during think aloud, and noted for future consideration.

#### Pregame and Postgame Cravings

Among these 30 smokers, measurement of QSU-Brief established a pregame mean of 3.24, (SD 1.65 on a 7-point Likert scale) and postgame mean of 2.99 (SD 1.40), a decrease of 0.25 points in cravings, which is a measurable but not significant difference (*P* =.11; Table 2). Across the ten individual items, two items had a significant improvement, “Smoking would make me less depressed” and “A cigarette would taste good now” (*P* =.03 and .002, respectively). As noted, estimating *P* values for each item represents an instance of multiple comparisons testing for a single research question. *P* values are presented “as-is” but should be considered measures of the relative strength of items, not definitive results.

**Table 2:** Pregame and postgame cravings of Phase 2 smokers.

Craving scale	Pregame		Postgame		Change (∆)	*P* value
	Mean	SD	Mean	SD		
Smoking would make me less depressed	3.36	1.93	2.7	1.51	-0.66	.03
A cigarette would taste good now	3.86	2.03	3.24	1.84	-0.62	.002
All I want now is a cigarette	2.86	1.81	2.56	1.56	-0.30	.28
I would do almost anything for a cigarette now	2.4	1.65	2.13	1.33	-0.27	.24
I could control things better right now if I could smoke	2.96	1.9	2.7	1.55	-0.26	.32
I have an urge for a cigarette	3.48	2.15	3.31	1.91	-0.17	.57
If it were possible, I would probably smoke now	3.8	1.98	3.73	1.85	-0.07	.77
I have a desire for a cigarette now	3.4	2.16	3.5	1.85	0.10	.75
Nothing will be better than smoking a cigarette now	2.86	1.97	3	1.44	0.14	.66
I am going to smoke as soon as possible	3.23	2.02	3.06	1.92	0.16	.32
Overall	3.24	1.65	2.99	1.40	-0.25	.11

In statistical analysis, the QSU-Brief decrease in prepost measurement was significant for those who quit for more than 48 hours (pregame mean 2.84, SD 1.16; postgame mean 2.0, SD 0.94; change 0.84; *P* = *.* 03). Cravings reduction was not significant for those smoking in last two days (pregame mean 3.14, SD 1.55; postgame mean 2.91, SD 1.06; change 0.23, *P* = *.* 24). Comparing those who reported that the game was challenging with those who did not, we observed a greater decrease in craving levels (pregame mean 3.26, SD 2.17; postgame mean 2.69, SD 1.66; change 0.57, *P* =.09; Table 3)

**Table 3:** Stratified analysis of prepost cravings of Phase 2 smokers.

QSU-Brief Question/Response		N	Pregame craving level Mean (SD)	Postgame craving level Mean (SD)				Change (∆)	*P* value
**I thought this game was fun**									
	Agree	22	3.3 (1.70)			2.96 (1.31)	- 0.32		.10
	Disagree	8	3.11 (1.61)			3.07 (1.71)	- 0.04		.10
**I thought the game was challenging **									
	Strongly agree	10	3.26 (2.17)			2.69 (1.66)	- 0.57		.09
	Others	19	3.08 (1.24)			2.97 (1.03)	- 0.11		.52
**It was a pleasant experience playing the game**									
	Agree	22	3.26 (1.70)			2.95 (1.30)	- 0.31		.11
	Disagree	7	3.48 (1.46)			3.35 (1.71)	- 0.13		.57
**This game would help me distract from cravings**									
	Agree	17	3.55 (1.98)			3.28 (1.63)	-0.27		.27
	Disagree	13	2.83 (1.01)			2.61 (0.96)	-0.22		.02
**Do you want to stop smoking?**									
	Abstinent >48 hours	5	2.84 (1.16)			2.0 (0.94)	-0.84		.03
	Yes	20	3.14 (1.55)			2.91 (1.06)	-0.23		.24
	No	5	4.04 (2.46)		4.32 (2.12)		+0.28		.29

### Phase 2 Dissemination Results

**Figure 3:**
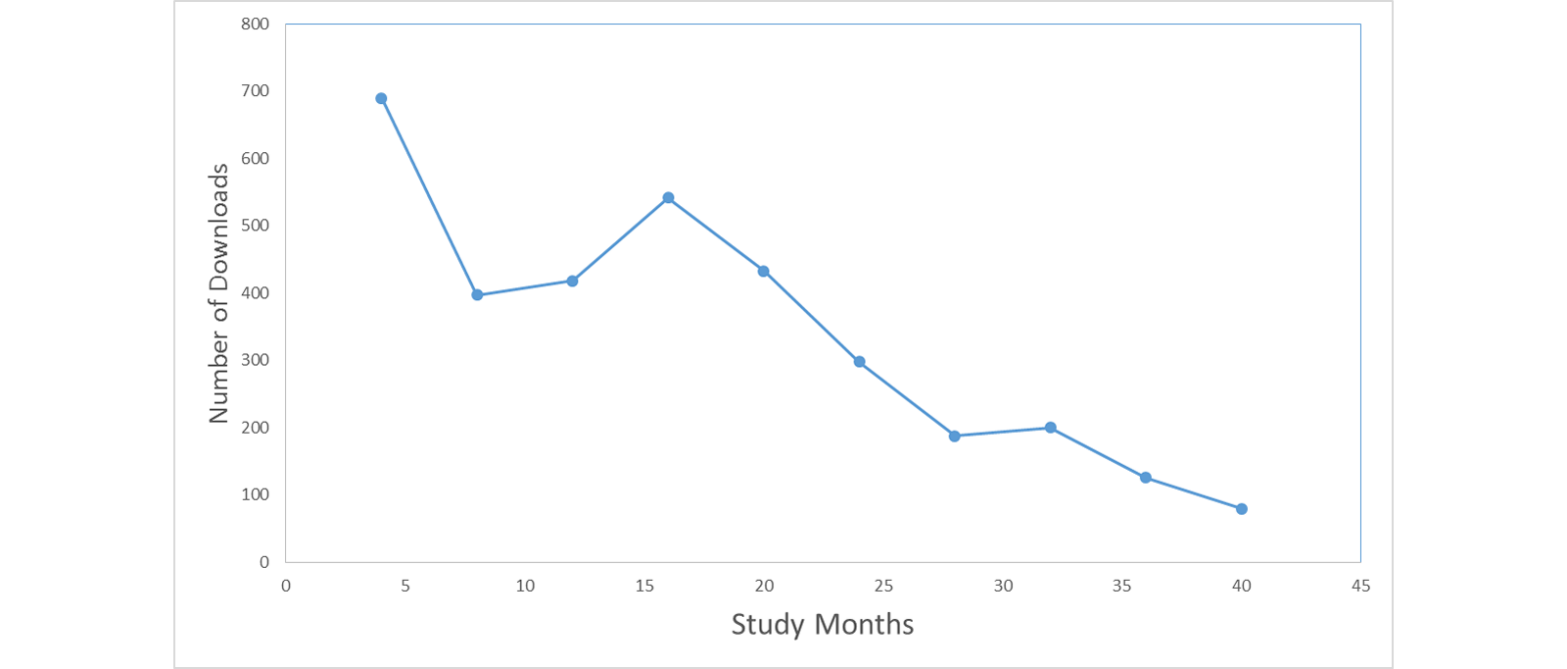
Number of downloads every quarter study period (December 2011 to June 2014).

Between December 22, 2011, and May 29, 2014, the game was downloaded 3372 times from the Apple iTunes App Store. As shown in Figure 3 , there was an initial spike in the number of downloads (690) and then again in quarter 4 (542). After quarter 4, the number of downloads tailed off. From January 25, 2012, to May 29, 2014, there were 1526 visits to the Decide2Quite.org Web site from the game, out of which 22 smokers registered on D2Q.

## Discussion

Our pilot study provides preliminary data on the potential of using a mobile game for cravings management, and demonstrates the challenges of developing games for health. Our two-phased approach was successful in pilot testing a game that was challenging and fun and reduced cravings. Smokers found the game enjoyable and that it would motivate them to quit smoking. Cravings reduction was significant in participants abstinent for more than 48 hours. Cravings reduction was also accentuated among those smokers who found the game challenging.

Research on games for smoking cessation is new [41]. Placing our evaluation in context of other studies, games are being developed to change smokers’ attitudes toward tobacco addiction to help smokers, and enhance their coping skills [42,43]. A qualitative study assessed the use of existing games (eg, Tetris) as part of a suite of tools to distract smokers from cravings [10]. To our knowledge, Crave-Out is the first mobile game developed and pilot tested specifically as a distraction/motivating game for managing smoking cravings.

Developing a health game is challenging, as game developers have to intermix evidence-based strategies with fun and game play elements [23,41,44]. While behavioral scientists are trained in incorporating evidence-based strategies in interventions, finding ways to intermix these strategies with something fun and game play requires new approaches. Adapting from game development practices, our prototype-based concept refinement approach allowed us to identify important issues early, before time and effort was spent in fully developing the game. For example, while we initially presented bad things associated with smoking, this had the unintended consequence of triggering the participants’ thoughts toward smoking. We were also able to make the game fun and challenging for our players by developing it as a multilevel game, with each level increasing in difficulty. Using multiple levels with increasing difficulty is a standard practice in computer games. The experience of facing and overcoming increasing difficulty has been shown to increase engagement and enjoyment of playing the game [23,41,44].

The reduction in cravings was accentuated in those participants abstinent for more than 48 hours. The reduction in cravings was also pronounced among smokers who found the game to be challenging. As noted, creating a game that is challenging may be key for distraction of cravings. Careful consideration should be used when attempting to balance “challenge” with “frustration” [23,41,44]. If the game is too challenging, it can lead to frustration, and trigger the smoker to smoke. If the game is not sufficiently challenging, it can lead to boredom. As noted above, we were able to identify strategies to increase the challenge element of the game, including designing the game as a pattern memory challenge game and the use of levels of increasing difficulty.

The majority of participants (53%) also responded that the game would motivate them to quit smoking. While any game might be distracting, having the ability to add motivational messages might represent the biggest advantage of creating a game specifically for smoking cessation. Adding tailored messages might have increased the motivational potential of our game. Tailored health messages can be highly effective in assisting individuals in understanding and responding to health concerns [45]. Future research is needed to further understand how to add these messages to a game without reducing the fun aspect of the game.

Engaging new smokers is an identified national priority in the State-of-the-Science Conference Statement on Tobacco Use [46], and our pilot study suggests that games might be a useful tool for this purpose. Without any advertisements, smokers found and downloaded our game from the Apple iTunes App Store. The game also engaged smokers to visit the Decide2Quit.org Web-assisted tobacco intervention. About half of the smokers who downloaded the game to the intervention also visited the Web site. Future studies are needed to test this engagement potential.

### Limitations

Limitations of this pilot study include a small sample size and a lack of physiological or biological assessments of participants’ pregame and postgame cravings levels. Selection bias is another potential limitation as participants were recruited through tobacco treatment specialists in a hospital setting and potential participants needed to be able to visit the usability laboratory to participate. The game may need to be made more accessible for smokers with different characteristics.Further research is needed to develop games that are easily accessed as distraction/motivation tools.

### Conclusions

Developing a distraction/motivation game to help smokers manage cravings is an iterative process. Using a 2-phased, user-testing approach helped us identify some important issues in game development. In a laboratory setting, our game resulted in reduced cravings, accentuated among those abstinent for more than 48 hours. Our next steps include a large study to assess craving management in a real-world setting.

## Appendix

Multimedia Appendix 1 Phase 1 Game Play.

Multimedia Appendix 2 Phase 2 Game Play.

## Abbreviations

FDA: Food and Drug Administration
GED: General Educational Development
QSU-Brief: Questionnaire of Smoking Urge-Brief
